# Supplementary material for: Comparison of Untargeted and Markers Analysis of Volatile Organic Compounds with SIFT-MS and SPME-GC-MS to Assess Tea Traceability
Source: Foods. 2024 Dec 11;13(24):3996. doi: 10.3390/foods13243996 (PMC11675271; doi:10.3390/foods13243996)
Supplement: Supplementary file 1 [file foods-13-03996-s001.zip › foods-3310566-supplementary.pdf]

Comparison of untargeted and markers analysis of volatile organic compounds with SIFT-MS and SPME-GC-MS to assess tea traceability.

Marine Reyrolle[1], Valerie Desauziers[1], Thierry Pigot[1], Lydia Gautier[2] and Mickael Le Bechec[1*]

[1] Universite de Pau et des Pays de l'Adour, E2S UPPA, CNRS, IMT Mines Ales, IPREM, Institut des sciences analytiques et de physicochimie pour l'environnement et les matériaux, UMR5254, Helioparc, 2 avenue President Angot, 64053, PAU cedex 9, France

[2] T Edition, 63 rue Vercingétorix, Paris, France

[*] Correspondence: mickael.lebechec@univ-pau.fr;

Supplementary Information

The HS-SPME-GC-MS-FID analysis were performed on a gas chromatograph (Agilent 7890B, Agilent, Santa Clara, CA, USA) coupled with an MS detector (Agilent 5977B) and a flame ionization detector and connected to a multi-function autosampler (Combi-Pal, CTC Analytics, Zwingen, Switzerland). Both MS and FID signals were used for this work. MS signals were used to identify compounds and for the untargeted analysis with Chromcompare + software. FID signal was used for pseudo-quantification of the 48 selected compounds.

SIFT-MS Voice 200 ultra was used in one hand in Multi-Ion-Monitoring mode to measure de concentration of VOCs in the head space (in ppbV). In the other hand, the SIFT-MS was also used with untargeted approach based on the raw signal (count.s^{-1}) corrected with the Internal Calibration Factor (ICF) daily determined with the calibration standard gas mixture. These signals were not converted into concentrations.

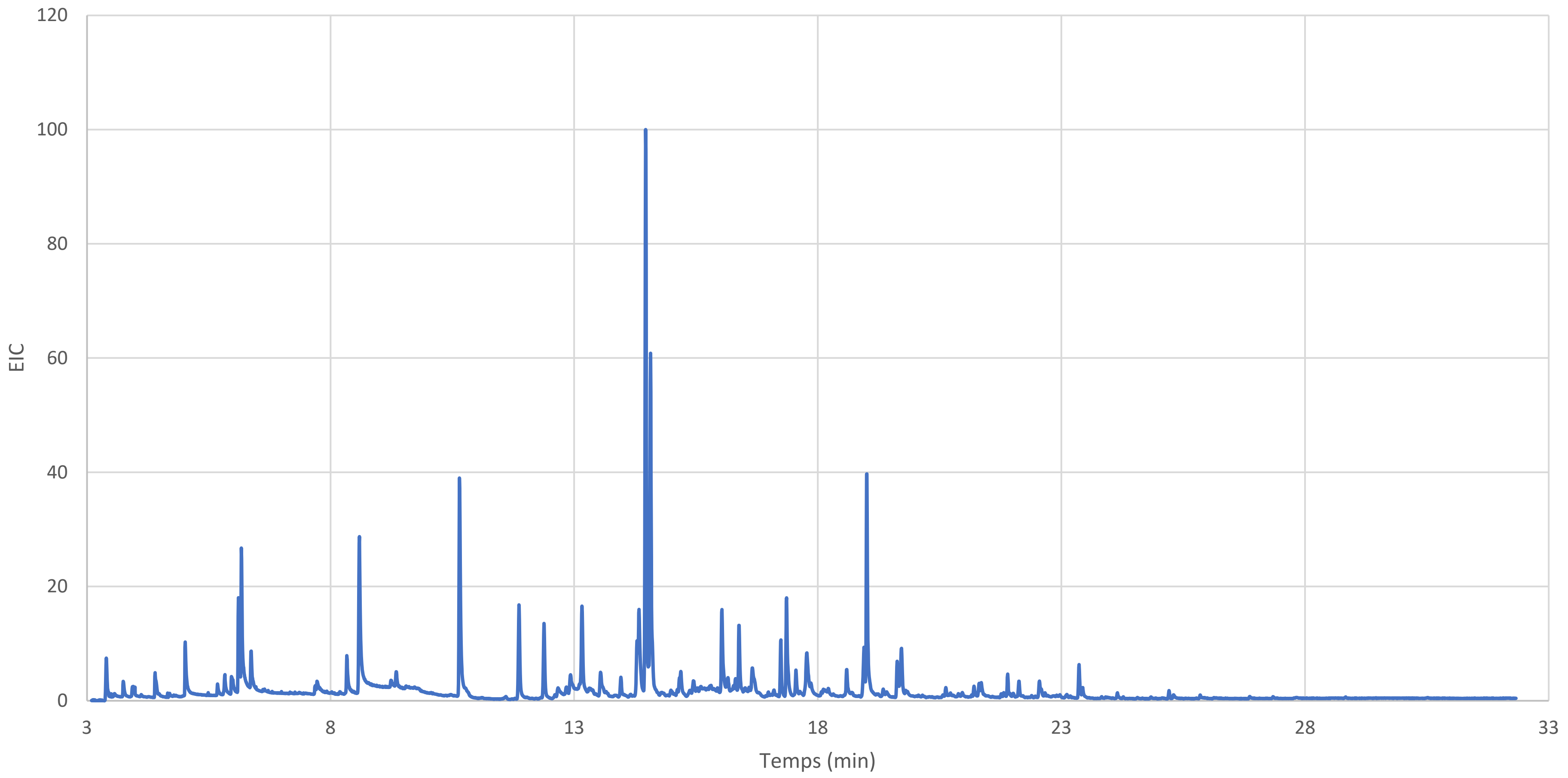

Figure S1: chromatogram of a HS-SPME-GC-MS analysis of tea sample.

Compounds	CAS	MM (g/mol)	Family	Discriminant for
**1-Penten-3-ol**	616-25-1	86.13	alcohol	▲ INDIA
**1-Pentanol**	71-41-0	88.15	alcohol	▲ INDIA
**2-Penten-1-ol**	1576-95-0	86.13	alcohol	▲ INDIA
**3-Hexen-1-ol**	544-12-7	100.16	alcohol	▲ INDIA
**2-Hexen-1-ol**	928-95-0	100.16	alcohol	✧ SRI LANKA
**1-Hexanol**	111-27-3	102.17	alcohol	☐ VIETNAM
**1-Octen-3-ol**	3391-86-4	128.21	alcohol	▲ INDIA
**Benzyl alcohol**	100-51-6	108.14	alcohol	▲ INDIA
**Linalool oxide**	5989-33-3	170.25	alcohol	▲ INDIA
**trans Linalool oxide**	34995-77-2	170.25	alcohol	▲ INDIA
**Phenylethyl alcohol**	60-12-8	122.16	alcohol	▲ INDIA
**trans-Linalool 3,7 oxide**	39028-58-5	170.25	alcohol	● CHINA
**beta-Myrcene**	123-35-3	136.23	terpene	✧ SRI LANKA
**D-Limonene**	5989-27-5	136.23	terpene	✧ SRI LANKA
**beta-cis-Ocimene**	3338-55-4	136.23	terpene	● CHINA
**Linalool**	78-70-6	154.25	terpene	▲ INDIA
**beat-Cyclocitral**	432-25-7	152.23	terpene	▲ INDIA
**Linalyl acetate**	115-95-7	196.29	terpene	▲ INDIA
**D-Carvone**	2244-16-8	150.22	terpene	☐ VIETNAM
**Geraniol**	106-24-1	154.25	terpene	× NEPAL
**alpha-Longipinene**	5989-08-2	204.35	terpene	✧ SRI LANKA
**beta-Ionone**	14901-07-6	192.3	terpene	▲ INDIA
**beta-Ionone epoxide**	23267-57-4	208.3	terpene	+ JAPAN
**Pentanal**	110-62-3	86.13	aldehyde	▲ INDIA
**Hexanal**	66-25-1	100.16	aldehyde	▲ INDIA
**2-Hexenal**	6728-26-3	98.14	aldehyde	× NEPAL
**Heptanal**	111-71-7	114.19	aldehyde	☐ VIETNAM
**Benzaldehyde**	100-52-7	106.12	aldehyde	× NEPAL
**Octanal**	124-13-0	128.21	aldehyde	● CHINA
**2,4-Heptadienal**	4313-03-5	110.15	aldehyde	☐ VIETNAM
**Nonanal**	124-19-6	142.24	aldehyde	☐ VIETNAM
**3-Penten-2-one, 4-methyl**	141-79-7	98.14	ketone	☐ VIETNAM
**2-Heptanone**	110-43-0	114.19	ketone	☐ VIETNAM
**Butyrolactone**	96-48-0	86.09	ketone	▲ INDIA
**5-Hepten-2-one, 6-methyl**	110-93-0	126.2	ketone	▲ INDIA
**3,5-Octadien-2-one**	30086-02-3	124.18	ketone	▲ INDIA
**Methyl salicylate**	119-36-8	152.15	ester	▲ INDIA
**Hexanoic acid, methyl ester**	106-70-7	130.18	ester	● CHINA
**Butanoic acid, 2-methyl-, hexyl ester**	10032-15-2	186.29	ester	☐ VIETNAM
**Decane**	124-18-5	142.28	alkane	▲ INDIA
**Undecane, 3-methyl**	1002-43-3	170.33	alkane	☐ VIETNAM
**Dodecane**	112-40-3	170.33	alkane	✧ SRI LANKA
**Tetradecane**	629-59-4	198.39	alkane	✧ SRI LANKA
**Acetic acid**	64-19-7	60.05	carboxylic acid	▲ INDIA
**Hexanoic acid**	142-62-1	116.16	carboxylic acid	▲ INDIA
**Butanoic acid, 4-hydroxy**	591-81-1	104.1	carboxylic acid	☐ VIETNAM
**Furan, 2-pentyl**	3777-69-3	138.21	furan	▲ INDIA
**Caffeine**	58-08-2	194.19	heterocyclic compound	▲ INDIA

Table S1 : list of the 48 selected compounds for targeted analysis of HS-SPME-GC-MS-FID analysis and their contribution to the supervised analysis (PLS-DA) according to the country.

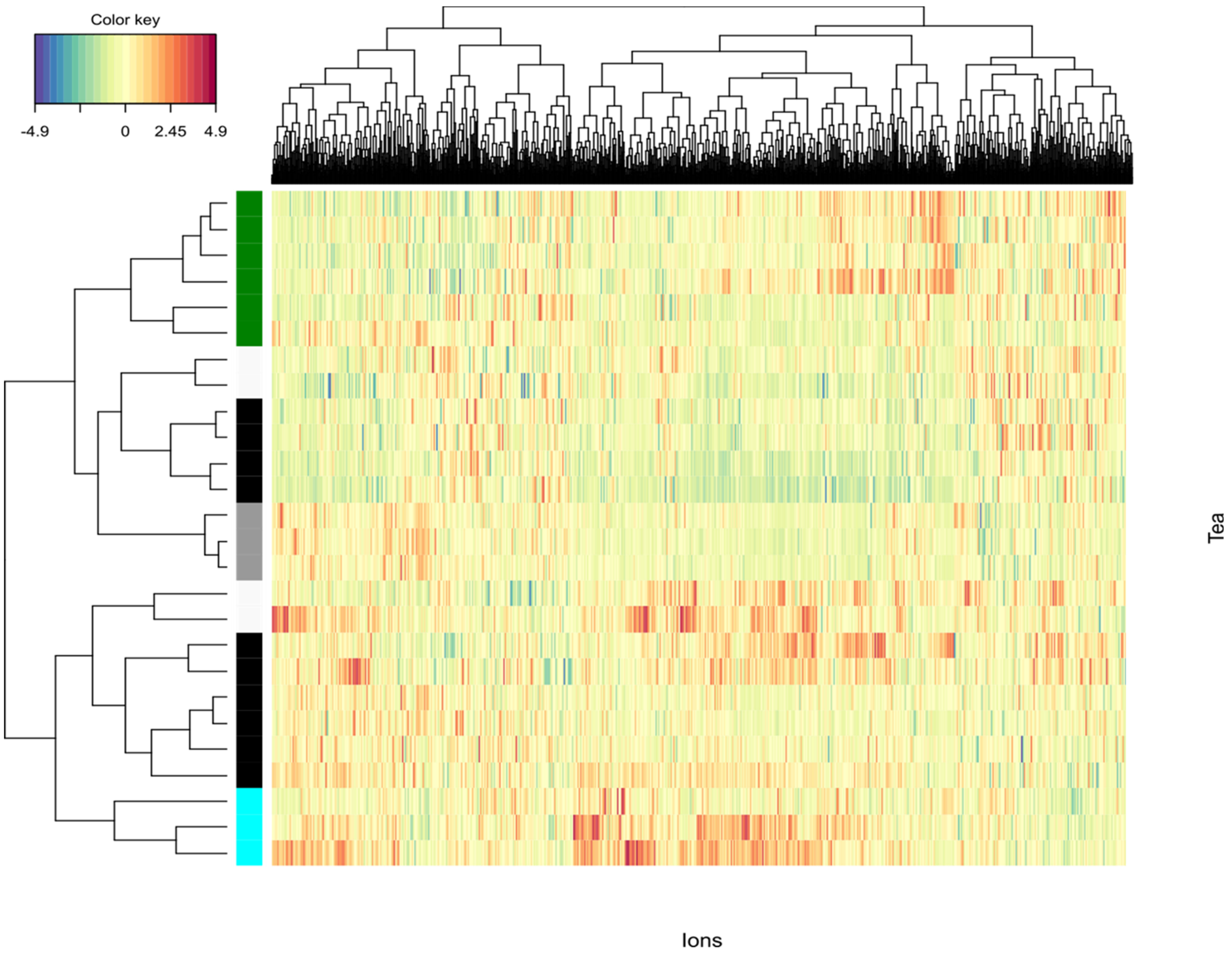

Figure S2: Heatmap of sPLS-DA of SIFT-MS fingerprints according to the color with a clustering of tea samples (horizontal) and a clustering of SIFT-MS ions (vertical)

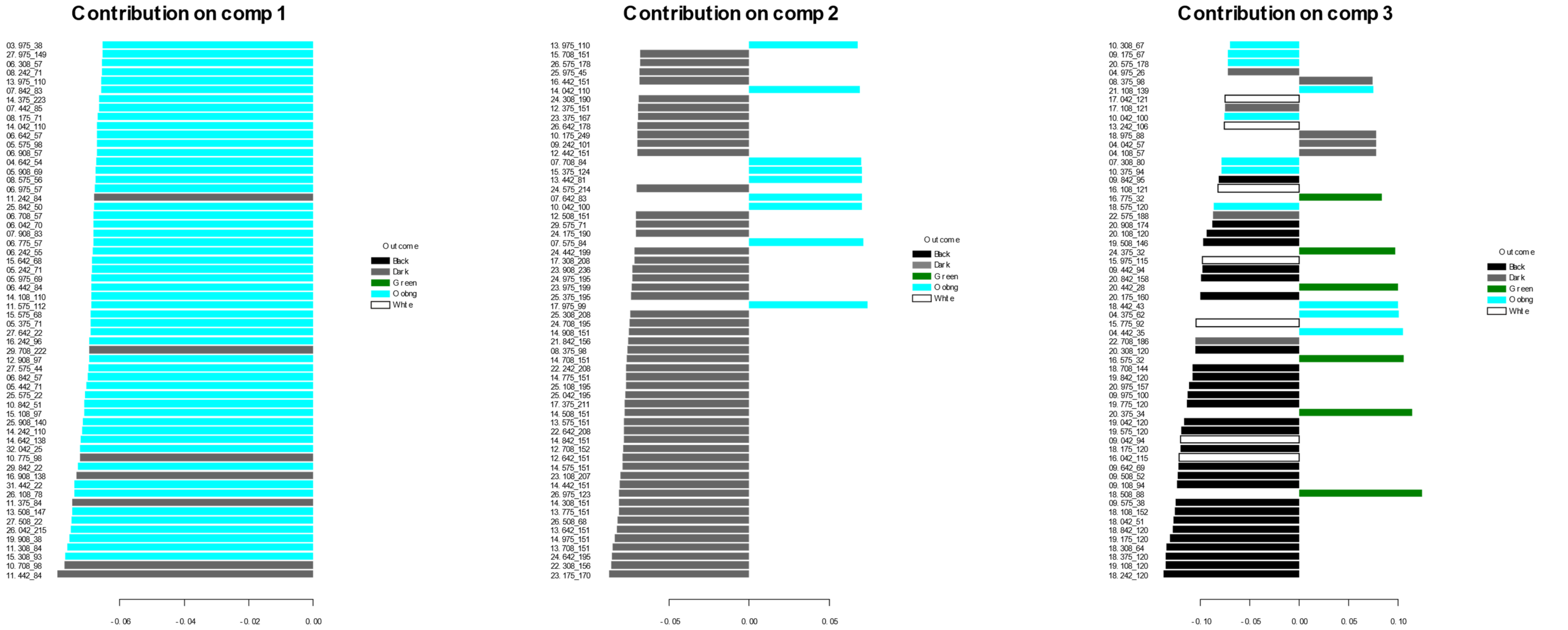

Figure S3: 60 most contributing variables of the sPLS-DA analysis on untargeted GC-MS measurements according to the color of tea (Component 1, component 2 and component 3). The variables code given with Chromcompare+ ® corresponds to the retention time in minute _ mass of the molecular ion (for example 11.442_84 corresponds to the retention time 11.442 minutes on aligned chromatograms and an ion with a mass on charge ratio of 84)

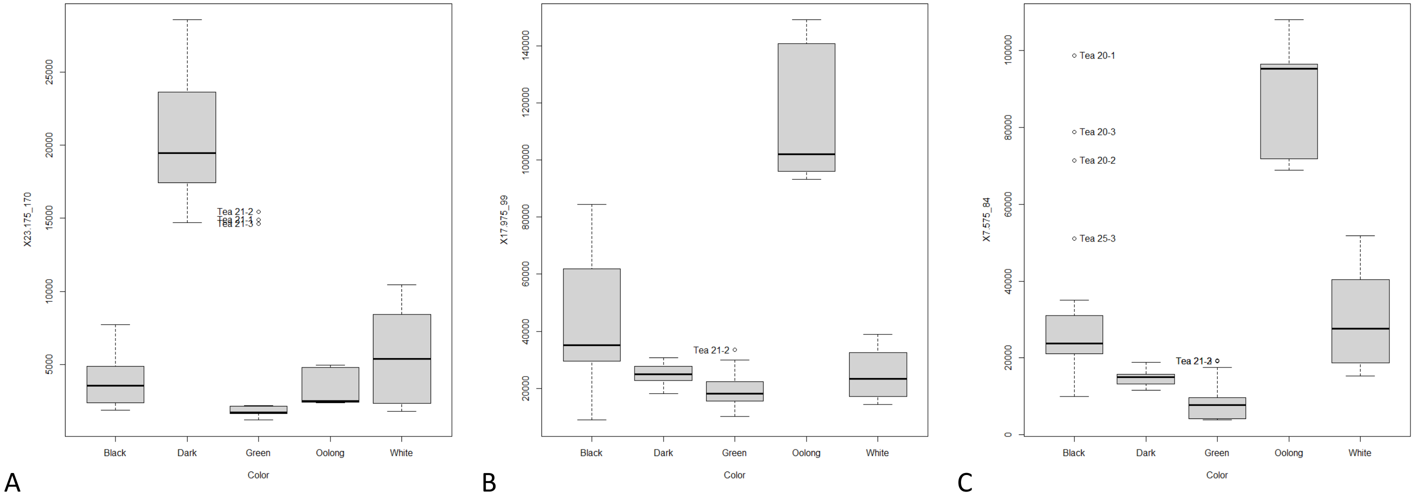

A B C

Figure S4: example of features values identified with sPLS-DA analysis on GC-MS measurements. A: component 23.175_170 significantly overexpressed in dark teas (retention time 23.175 minutes and pseudo molecular ion m/z 170). This compound corresponds to Dihydroactinidiolide, a volatile terpene with a NISTDB correspondence's score of 90.3%. B: component 17.975_99 significantly overexpressed in dark teas (retention time 17.975 minutes and pseudo molecular ion m/z 99). C: component 7.575_84 significantly overexpressed in oolong teas (retention time 7.575 minutes and pseudo molecular ion m/z 84). These two last features were not linked to identified compounds because of co-elutions.

Table S2: Labsyft (release 1.6.2 SYFT®) MIM method for the quantification of the 28 known tea markers.

A: Compounds selection

TeaMarkersSIM.sme - LabSyft Method Editor

File Options Help

carrier

Information | Settings | Compound Selection | Compound Calculation | Scanned Masses

	Compound	Safe limit (ppb)	Reporting threshold (ppb)	Tolerance ratio (%)	Subtract	Hide	Significant digits	Decimal places
He	1-penten-3-ol			20	☐	☐	3	
He	1-pentanol			20	☐	☐	3	
He	cis-2-penten-1-ol			20	☐	☐	3	
He	3-hexen-1-ol			20	☐	☐	3	
He	(E)-2-hexen-1-ol			20	☐	☐	3	
He	1-hexanol			20	☐	☐	3	
He	1-octen-3-ol			20	☐	☐	3	
He	benzyl alcohol			20	☐	☐	3	
He	2-phenylethanol			20	☐	☐	3	
He	myrcene			20	☐	☐	3	
He	(R)-limonene			20	☐	☐	3	
He	(Z)-beta-ocimene			20	☐	☐	3	
He	linalool			20	☐	☐	3	
He	beta-cyclocitral			20	☐	☐	3	
He	linalyl acetate			20	☐	☐	3	
He	D-carvone			20	☐	☐	3	
He	lemonol			20	☐	☐	3	
He	beta-ionone			20	☐	☐	3	
He	pentanal			20	☐	☐	3	
He	hexanal			20	☐	☐	3	
He	(E)-2-hexenal			20	☐	☐	3	
He	heptanal			20	☐	☐	3	
N2	benzaldehyde			20	☐	☐	3	
He	octanal			20	☐	☐	3	
He	2,4-heptadienal			20	☐	☐	3	
He	nonanal			20	☐	☐	3	
He	2-heptanone			20	☐	☐	3	
He	1,4-butyrolactone			20	☐	☐	3	
He	6-methyl-5-hepten-2-one			20	☐	☐	3	
He	methyl salicylate			20	☐	☐	3	
He	methyl hexanoate			20	☐	☐	3	
He	decane			20	☐	☐	3	
He	dodecane			20	☐	☐	3	
He	tetradecane			20	☐	☐	3	
He	acetic acid			20	☐	☐	3	
He	hexanoic acid			20	☐	☐	3	
He	2-pentylfuran			20	☐	☐	3	

B: product ions corresponding to the compounds. *Underligned ions correspond to conflict ions*

TeaMarkersSIM.sme - LabSyft Method Editor

File Options Help

carrier

Information | Settings | Compound Selection | Compound Calculation | Scanned Masses

Show all | Compound: All | Reaction rate: All | ☑ Secondary | ○ Dry | ◉ W

Reagent: All | Branching ratio: All

Warning: All products

Compound	Reagent	Reaction rate	Branching ratio (%)	Mass (m/z)	Product	Scan	Calculate
1-penten-3-ol	H3O+	2.6E-9	4	45	C2H5O+	☐	☐
2,4-heptadienal	H3O+	3.0E-9	10	47	C2H7O+	☐	☐
(Z)-beta-ocimene	H3O+	2.6E-9	3	57	C4H9+	☐	☐
linalyl acetate	H3O+	3.0E-9	75	59	C2H3O2+	☐	☐
2,4-heptadienal	H3O+	3.0E-9	85	59	C3H7O+	☐	☐
2,4-heptadienal	H3O+	3.0E-9		77	C3H7O.H2O+	☑	☑
acetic acid	H3O+	2.6E-9	100	61	CH3COOH2+	☑	☑
acetic acid	H3O+	2.6E-9		79	CH3COOH2+.H2O	☑	☑
acetic acid	H3O+	2.6E-9		97	CH3COOH2+.2H2O	☐	☐
(Z)-beta-ocimene	H3O+	2.6E-9	3	69	C5H9+	☐	☐
1-octen-3-ol	H3O+	2.5E-9	8	69	C5H9+	☐	☐
1-penten-3-ol	H3O+	2.6E-9	85	69	C5H9+	☐	☐
cis-2-penten-1-ol	H3O+	3.0E-9	100	69	C5H9+	☐	☐
myrcene	H3O+	2.6E-9	3	69	C5H9+	☐	☐
pentanal	H3O+	3.6E-9	35	69	C5H9+	☐	☐
1-pentanol	H3O+	2.8E-9	100	71	C5H11+	☑	☑
(R)-limonene	H3O+	2.6E-9	29	81	C6H9+	☐	☐
(Z)-beta-ocimene	H3O+	2.6E-9	24	81	C6H9+	☐	☐
linalool	H3O+	3.1E-9	35	81	C6H9+	☐	☐
linalyl acetate	H3O+	3.0E-9	10	81	C6H9+	☐	☐
myrcene	H3O+	2.6E-9	30	81	C6H9+	☐	☐
(E)-2-hexen-1-ol	H3O+	3.3E-9	100	83	C6H11+	☐	☐
3-hexen-1-ol	H3O+	3.2E-9	65	83	C6H11+	☐	☐
hexanal	H3O+	3.7E-9	50	83	C6H11+	☐	☐
1-hexanol	H3O+	2.9E-9	100	85	C6H13+	☑	☑
1,4-butyrolactone	H3O+	4.1E-9	100	87	C4H7O2+	☐	☐
1,4-butyrolactone	H3O+	4.1E-9		105	C4H7O2+.H2O	☐	☐
1,4-butyrolactone	H3O+	4.1E-9		123	C4H7O2+.2H2O	☐	☐
1-penten-3-ol	H3O+	2.6E-9	11	87	C5H10O.H+	☐	☐
1-penten-3-ol	H3O+	2.6E-9		105	C5H10O.H+.H2O	☐	☐
pentanal	H3O+	3.6E-9	65	87	C5H11O+	☐	☐
pentanal	H3O+	3.6E-9		105	C5H11O+.H2O	☐	☐
pentanal	H3O+	3.6E-9		123	C5H11O+.2H2O	☐	☐
benzyl alcohol	H3O+	2.8E-9	100	91	C7H7+	☑	☑
(R)-limonene	H3O+	2.6E-9	3	95	C7H11+	☐	☐
(Z)-beta-ocimene	H3O+	2.6E-9	4	95	C7H11+	☐	☐
linalool	H3O+	3.1E-9	5	95	C7H11+	☐	☐
myrcene	H3O+	2.6E-9	9	95	C7H11+	☐	☐
heptanal	H3O+	3.7E-9	20	97	C7H13+	☐	☐
methyl hexanoate	H3O+	3.0E-9	8	99	C6H10O.H+	☐	☐
(E)-2-hexenal	H3O+	4.6E-9	100	99	C6H11O+	☐	☐
(E)-2-hexenal	H3O+	4.6E-9		117	C6H11O+.H2O	☐	☐
(E)-2-hexenal	H3O+	4.6E-9		135	C6H11O+.2H2O	☐	☐
hexanoic acid	H3O+	3.0E-9	25	99	C6H11O+	☐	☐
3-hexen-1-ol	H3O+	3.2E-9	35	101	C6H12O.H+	☐	☐
3-hexen-1-ol	H3O+	3.2E-9		119	C6H12O.H+.H2O	☐	☐
hexanal	H3O+	3.7E-9	50	101	C6H13O+	☐	☐

hexanal	H3O+	3.7E-9		119	C6H13O+.H2O	☐	☐
hexanal	H3O+	3.7E-9		137	C6H13O+.2H2O	☐	☐
2-phenylethanol	H3O+	2.9E-9	100	105	C8H9+	☐	☐
2-phenylethanol	H3O+	2.9E-9		123	C8H9+.H2O	☐	☐
2-phenylethanol	H3O+	2.9E-9		141	C8H9+.2H2O	☑	☑
benzaldehyde	H3O+	3.7E-9	100	107	C7H7O+	☑	☑
benzaldehyde	H3O+	3.7E-9		125	C7H7O+.H2O	☐	☐
benzaldehyde	H3O+	3.7E-9		143	C7H7O+.2H2O	☐	☐
6-methyl-5-hepten-2-one	H3O+	3.0E-9	50	109	C8H13+	☑	☑
2,4-heptadienal	H3O+	3.0E-9	5	111	C7H10O.H+	☐	☐
1-octen-3-ol	H3O+	2.5E-9	83	111	C8H15+	☐	☐
octanal	H3O+	3.8E-9	15	111	C8H15+	☐	☐
2-heptanone	H3O+	4.0E-9	100	115	C7H14OH+	☐	☐
2-heptanone	H3O+	4.0E-9		133	C7H14OH+.H2O	☐	☐
heptanal	H3O+	3.7E-9	80	115	C7H15O+	☐	☐
heptanal	H3O+	3.7E-9		133	C7H15O+.H2O	☐	☐
heptanal	H3O+	3.7E-9		151	C7H15O+.2H2O	☐	☐
hexanoic acid	H3O+	3.0E-9	75	117	C6H12O2.H+	☐	☐
beta-cyclocitral	H3O+	3.0E-9	5	123	C8H10O.H+	☐	☐
nonanal	H3O+	2.5E-9	14	125	C9H17+	☐	☐
6-methyl-5-hepten-2-one	H3O+	3.0E-9	50	127	C8H14O.H+	☑	☑
1-octen-3-ol	H3O+	2.5E-9	9	129	C8H16O.H+	☐	☐
1-octen-3-ol	H3O+	2.5E-9		147	C8H16O.H+.H2O	☐	☐
octanal	H3O+	3.8E-9	85	129	C8H17O+	☐	☐
octanal	H3O+	3.8E-9		147	C8H17O+.H2O	☐	☐
octanal	H3O+	3.8E-9		165	C8H17O+.2H2O	☑	☑
methyl hexanoate	H3O+	3.0E-9	92	131	C7H14O2.H+	☑	☑
(R)-limonene	H3O+	2.6E-9	68	137	C10H17+	☐	☐
(Z)-beta-ocimene	H3O+	2.6E-9	66	137	C10H17+	☐	☐
lemonol	H3O+	3.1E-9	95	137	C10H17+	☐	☐
linalool	H3O+	3.1E-9	55	137	C10H17+	☐	☐
linalyl acetate	H3O+	3.0E-9	15	137	C10H17+	☐	☐
myrcene	H3O+	2.6E-9	58	137	C10H17+	☐	☐
2-pentylfuran	H3O+	3.0E-9	100	139	C9H14O.H+	☑	☑
nonanal	H3O+	2.5E-9	86	143	C9H19O+	☐	☐
D-carvone	H3O+	3.4E-9	100	151	C10H14O.H+	☐	☐
D-carvone	H3O+	3.4E-9		169	C10H14O.H3O+	☑	☑
beta-cyclocitral	H3O+	3.0E-9	95	153	C10H16O.H+	☐	☐
methyl salicylate	H3O+	4.5E-9	100	153	C8H8O3H+	☐	☐
lemonol	H3O+	3.1E-9	5	155	C10H18O.H+	☐	☐
linalool	H3O+	3.1E-9	5	155	C10H18O.H+	☐	☐
decane	H3O+	1.6E-9	100	161	H3O+.C10H22	☑	☑
dodecane	H3O+	2.8E-9	100	189	H3O+.C12H26	☑	☑
beta-ionone	H3O+	3.0E-9	100	193	C13H20O.H+	☑	☑
beta-ionone	H3O+	3.0E-9		211	C13H20O.H+.H2O	☑	☑
tetradecane	H3O+	2.9E-9	100	217	C14H30.H3O+	☑	☑
2,4-heptadienal	NO+	2.1E-9	5	45	C2H5O+	☐	☐

		rate	ratio (%)	(m/z)			
1-penten-3-ol	NO+	2.4E-9	5	57	C3H5O+	☐	☐
2,4-heptadienal	NO+	2.1E-9	80	57	C3H5O+	☐	☐
cis-2-penten-1-ol	NO+	2.7E-9	4	68	C5H8+	☐	☐
1-octen-3-ol	NO+	2.3E-9	2	69	C4H5O+	☐	☐
1-pentanol	NO+	2.5E-9	10	69	C5H9+	☐	☐
1-penten-3-ol	NO+	2.4E-9	37	69	C5H9+	☐	☐
cis-2-penten-1-ol	NO+	2.7E-9	24	69	C5H9+	☐	☐
(E)-2-hexenal	NO+	3.8E-9	15	71	C4H7O+	☐	☐
1-pentanol	NO+	2.5E-9	5	71	C5H11+	☐	☐
3-hexen-1-ol	NO+	2.9E-9	5	71	C5H11+	☐	☐
3-hexen-1-ol	NO+	2.9E-9	5	72	C4H8O+	☐	☐
linalyl acetate	NO+	1.0E-9	10	80	C6H8+	☐	☐
3-hexen-1-ol	NO+	2.9E-9	25	82	C6H10+	☐	☐
(E)-2-hexen-1-ol	NO+	2.9E-9	31	83	C6H11+	☑	☑
1-penten-3-ol	NO+	2.4E-9	56	85	C5H9O+	☐	☐
cis-2-penten-1-ol	NO+	2.7E-9	68	85	C5H9O+	☐	☐
pentanal	NO+	3.0E-9	100	85	C5H9O+	☐	☐
cis-2-penten-1-ol	NO+	2.7E-9	4	86	C5H10O+	☐	☐
1-pentanol	NO+	2.5E-9	85	87	C5H11O+	☑	☑
linalyl acetate	NO+	1.0E-9	65	88	C4H8O2+	☑	☑
acetic acid	NO+	9.0E-10	100	90	NO+.CH3COOH	☑	☑
acetic acid	NO+	9.0E-10		108	NO+.CH3COOH.H2O	☐	☐
benzyl alcohol	NO+	2.3E-9	10	91	C7H7+	☐	☐
(R)-limonene	NO+	2.2E-9	3	92	C7H8+	☐	☐
(Z)-beta-ocimene	NO+	2.1E-9	29	92	C7H8+	☐	☐
linalool	NO+	2.6E-9	5	92	C7H8+	☐	☐
myrcene	NO+	2.2E-9	11	92	C7H8+	☐	☐
(R)-limonene	NO+	2.2E-9	1	93	C7H9+	☐	☐
(Z)-beta-ocimene	NO+	2.1E-9	16	93	C7H9+	☐	☐
myrcene	NO+	2.2E-9	34	93	C7H9+	☐	☐
(R)-limonene	NO+	2.2E-9	2	94	C7H10+	☐	☐
linalool	NO+	2.6E-9	20	96	C7H12+	☑	☑
(E)-2-hexenal	NO+	3.8E-9	85	97	C6H9O+	☑	☑
(E)-2-hexen-1-ol	NO+	2.9E-9	55	99	C6H11O+	☐	☐
(E)-2-hexen-1-ol	NO+	2.9E-9		117	C6H11O+.H2O	☐	☐
1-octen-3-ol	NO+	2.3E-9	7	99	C6H11O+	☐	☐
3-hexen-1-ol	NO+	2.9E-9	50	99	C6H11O+	☐	☐
3-hexen-1-ol	NO+	2.9E-9		117	C6H11O+.H2O	☐	☐
hexanal	NO+	2.5E-9	100	99	C6H11O+	☐	☐
hexanoic acid	NO+	2.5E-9	10	99	C6H11O+	☐	☐
methyl hexanoate	NO+	2.1E-9	70	99	C6H11O+	☐	☐
(E)-2-hexen-1-ol	NO+	2.9E-9	10	100	C6H12O+	☐	☐
(E)-2-hexen-1-ol	NO+	2.9E-9		118	C6H12O+.H2O	☐	☐
3-hexen-1-ol	NO+	2.9E-9	15	100	C6H12O+	☐	☐
3-hexen-1-ol	NO+	2.9E-9		118	C6H12O+.H2O	☐	☐
1-hexanol	NO+	2.4E-9	100	101	C6H13O+	☑	☑
benzaldehyde	NO+	2.3E-9	100	105	C7H5O+	☑	☑

benzyl alcohol	NO+	2.3E-9	40	107	C7H7O+	☑	☑
benzyl alcohol	NO+	2.3E-9	50	108	C7H8O+	☐	☐
6-methyl-5-hepten-2-one	NO+	2.5E-9	40	108	C8H12+	☐	☐
2,4-heptadienal	NO+	2.1E-9	15	109	C7H9O+	☑	☑
linalool	NO+	2.6E-9	7	111	C7H11O+	☐	☐
1-octen-3-ol	NO+	2.3E-9	29	111	C8H15+	☐	☐
heptanal	NO+	3.3E-9	100	113	C7H13O+	☑	☑
1,4-butyrolactone	NO+	3.5E-9	100	116	C4H6O2.NO+	☑	☑
2-phenylethanol	NO+	2.3E-9	10	121	C8H9O+	☐	☐
(R)-limonene	NO+	2.2E-9	2	121	C9H13+	☐	☐
2-phenylethanol	NO+	2.3E-9	90	122	C8H10O+	☑	☑
6-methyl-5-hepten-2-one	NO+	2.5E-9	60	126	C8H14O+	☑	☑
1-octen-3-ol	NO+	2.3E-9	57	127	C8H15O+	☐	☐
octanal	NO+	3.0E-9	100	127	C8H15O+	☐	☐
(R)-limonene	NO+	2.2E-9	4	135	C10H15+	☐	☐
(R)-limonene	NO+	2.2E-9	88	136	C10H16+	☐	☐
(Z)-beta-ocimene	NO+	2.1E-9	55	136	C10H16+	☐	☐
linalool	NO+	2.6E-9	60	136	C10H16+	☐	☐
linalyl acetate	NO+	1.0E-9	25	136	C10H16+	☐	☐
myrcene	NO+	2.2E-9	55	136	C10H16+	☐	☐
lemonol	NO+	2.5E-9	40	137	C10H17+	☑	☑
2-pentylfuran	NO+	2.0E-9	100	138	C9H14O+	☑	☑
decane	NO+	1.5E-9	90	141	C10H21+	☐	☐
nonanal	NO+	2.7E-9	100	141	C9H17O+	☐	☐
2-heptanone	NO+	3.4E-9	100	144	C7H14O.NO+	☑	☑
hexanoic acid	NO+	2.5E-9	90	146	C6H12O2.NO+	☑	☑
D-carvone	NO+	2.8E-9	8	150	C10H14O+	☐	☐
beta-cyclocitral	NO+	3.0E-9	90	151	C10H15O+	☑	☑
methyl salicylate	NO+	2.1E-9	100	152	C8H8O3+	☑	☑
lemonol	NO+	2.5E-9	60	154	C10H18O+	☐	☐
linalool	NO+	2.6E-9	8	154	C10H18O+	☐	☐
methyl hexanoate	NO+	2.1E-9	30	160	C7H14O2.NO+	☐	☐
beta-cyclocitral	NO+	3.0E-9	10	168	C10H14O.H2O+	☐	☐
dodecane	NO+	1.5E-9	90	169	C12H25+	☑	☑
beta-ionone	NO+	2.5E-9	5	177	C12H17O+	☐	☐
D-carvone	NO+	2.8E-9	92	180	C10H14O.NO+	☑	☑
beta-ionone	NO+	2.5E-9	95	192	C13H20O+	☑	☑
1,4-butyrolactone	O2+	3.9E-9	80	42	C2H2O+ or C3H6+	☐	☐
1,4-butyrolactone	O2+	3.9E-9		60	C2H3O+.H2O or C3H6+.H2O	☐	☐
1-hexanol	O2+	2.6E-9	20	42	C3H6+	☐	☐
1-pentanol	O2+	2.8E-9	35	42	C3H6+	☐	☐
2-heptanone	O2+	3.2E-9	5	43	C2H3O+	☐	☐
(E)-2-hexenal	O2+	3.7E-9	30	43	C2H3O+ or C3H7+	☐	☐
1-hexanol	O2+	2.6E-9	10	43	C3H7+	☐	☐
linalyl acetate	O2+	2.9E-9	65	43	C3H7+	☐	☐
nonanal	O2+	1.5E-9	6	43	C3H7+	☐	☐
acetic acid	O2+	2.3E-9	50	43	CH3CO+	☐	☐

(E)-2-hexen-1-ol	O2+	2.9E-9	6	44	C2H4O+	☐	☐
cis-2-penten-1-ol	O2+	2.7E-9	7	44	C2H4O+	☐	☐
heptanal	O2+	3.2E-9	16	44	C2H4O+	☐	☐
hexanal	O2+	2.0E-9	32	44	C2H4O+	☐	☐
pentanal	O2+	1.5E-9	60	44	C2H4O+	☐	☐
2,4-heptadienal	O2+	2.1E-9	10	45	C2H5O+	☐	☐
1-pentanol	O2+	2.8E-9	10	55	C4H7+	☐	☐
(E)-2-hexen-1-ol	O2+	2.9E-9	5	56	C3H4O+	☐	☐
cis-2-penten-1-ol	O2+	2.7E-9	3	56	C3H4O+	☐	☐
1-hexanol	O2+	2.6E-9	40	56	C4H8+	☐	☐
hexanal	O2+	2.0E-9	53	56	C4H8+	☐	☐
(E)-2-hexen-1-ol	O2+	2.9E-9	44	57	C3H5O+	☐	☐
1-octen-3-ol	O2+	2.3E-9	28	57	C3H5O+	☐	☐
1-penten-3-ol	O2+	2.4E-9	75	57	C3H5O+	☐	☐
2,4-heptadienal	O2+	2.1E-9	40	57	C3H5O+	☐	☐
cis-2-penten-1-ol	O2+	2.7E-9	56	57	C3H5O+	☐	☐
hexanal	O2+	2.0E-9	5	57	C3H5O+	☐	☐
2-heptanone	O2+	3.2E-9	15	57	C4H9+	☐	☐
nonanal	O2+	1.5E-9	6	57	C4H9+	☐	☐
linalyl acetate	O2+	2.9E-9	20	58	C2H2O2+	☐	☐
2,4-heptadienal	O2+	2.1E-9	45	58	C3H6O+	☐	☐
2-heptanone	O2+	3.2E-9	50	58	C3H6O+	☐	☐
pentanal	O2+	1.5E-9	40	58	C3H6O+	☐	☐
acetic acid	O2+	2.3E-9	50	60	CH3COOH+	☐	☐
acetic acid	O2+	2.3E-9		61	CH3COOH.H+	☐	☐
acetic acid	O2+	2.3E-9		79	CH3COOH.H+.H2O	☐	☐
acetic acid	O2+	2.3E-9		97	CH3COOH.H+.2H2O	☐	☐
(E)-2-hexen-1-ol	O2+	2.9E-9	5	67	C5H7+	☐	☐
3-hexen-1-ol	O2+	2.9E-9	20	67	C5H7+	☐	☐
(R)-limonene	O2+	2.2E-9	10	68	C5H8+	☐	☐
(Z)-beta-ocimene	O2+	1.9E-9	4	68	C5H8+	☐	☐
1-octen-3-ol	O2+	2.3E-9	4	68	C5H8+	☐	☐
cis-2-penten-1-ol	O2+	2.7E-9	15	68	C5H8+	☐	☐
nonanal	O2+	1.5E-9	5	68	C5H8+	☐	☐
octanal	O2+	2.8E-9	10	68	C5H8+	☐	☐
3-hexen-1-ol	O2+	2.9E-9	10	69	C5H9+	☐	☐
lemonol	O2+	2.5E-9	75	69	C5H9+	☐	☐
myrcene	O2+	2.2E-9	9	69	C5H9+	☐	☐
(E)-2-hexenal	O2+	3.7E-9	30	69	C5H9+ or C4H5O+	☐	☐
(E)-2-hexenal	O2+	3.7E-9	20	70	C4H6O+	☐	☐
3-hexen-1-ol	O2+	2.9E-9	10	70	C4H6O+	☐	☐
1-hexanol	O2+	2.6E-9	10	70	C5H10+	☐	☐
1-pentanol	O2+	2.8E-9	45	70	C5H10+	☐	☐
heptanal	O2+	3.2E-9	38	70	C5H10+	☐	☐
nonanal	O2+	1.5E-9	4	70	C5H10+	☐	☐
(E)-2-hexen-1-ol	O2+	2.9E-9	7	71	C4H7O+	☐	☐
1-octen-3-ol	O2+	2.3E-9	3	71	C4H7O+	☐	☐

1-octen-3-ol	O2+	2.3E-9	3	71	C4H7O+	☐	☐
1-penten-3-ol	O2+	2.4E-9	2	71	C4H7O+	☐	☐
cis-2-penten-1-ol	O2+	2.7E-9	4	71	C4H7O+	☐	☐
heptanal	O2+	3.2E-9	12	71	C4H7O+	☐	☐
1-pentanol	O2+	2.8E-9	10	71	C5H11+	☐	☐
2-heptanone	O2+	3.2E-9	5	71	C5H11+	☐	☐
nonanal	O2+	1.5E-9	5	71	C5H11+	☐	☐
(E)-2-hexen-1-ol	O2+	2.9E-9	4	72	C4H8O+	☐	☐
1-octen-3-ol	O2+	2.3E-9	24	72	C4H8O+	☐	☐
nonanal	O2+	1.5E-9	5	72	C4H8O+	☐	☐
hexanal	O2+	2.0E-9	5	72	C5H12+	☐	☐
hexanal	O2+	2.0E-9		90	C5H12.H2O+	☐	☐
methyl hexanoate	O2+	1.7E-9	55	74	C4H10O+	☑	☑
benzyl alcohol	O2+	2.3E-9	20	79	C6H7+	☐	☐
(R)-limonene	O2+	2.2E-9	5	80	C6H8+	☐	☐
(Z)-beta-ocimene	O2+	1.9E-9	6	80	C6H8+	☐	☐
linalool	O2+	2.5E-9	20	80	C6H8+	☐	☐
linalyl acetate	O2+	2.9E-9	5	80	C6H8+	☐	☐
myrcene	O2+	2.2E-9	3	80	C6H8+	☐	☐
1-octen-3-ol	O2+	2.3E-9	3	81	C6H9+	☐	☐
2,4-heptadienal	O2+	2.1E-9	5	81	C6H9+	☐	☐
D-carvone	O2+	2.7E-9	20	81	C6H9+	☐	☐
nonanal	O2+	1.5E-9	4	81	C6H9+	☐	☐
(E)-2-hexen-1-ol	O2+	2.9E-9	15	82	C6H10+	☐	☐
1-octen-3-ol	O2+	2.3E-9	4	82	C6H10+	☐	☐
3-hexen-1-ol	O2+	2.9E-9	50	82	C6H10+	☐	☐
nonanal	O2+	1.5E-9	9	82	C6H10+	☐	☐
octanal	O2+	2.8E-9	15	82	C6H10+	☐	☐
3-hexen-1-ol	O2+	2.9E-9	5	83	C6H11+	☐	☐
linalool	O2+	2.5E-9	10	83	C6H11+	☐	☐
1-hexanol	O2+	2.6E-9	20	84	C6H12+	☐	☐
octanal	O2+	2.8E-9	45	84	C6H12+	☐	☐
1-octen-3-ol	O2+	2.3E-9	7	85	C5H9O+	☐	☐
nonanal	O2+	1.5E-9	6	85	C5H9O+	☐	☐
2-heptanone	O2+	3.2E-9	5	85	C6H13+	☐	☐
octanal	O2+	2.8E-9	10	85	C6H13+	☐	☐
1,4-butyrolactone	O2+	3.9E-9	20	86	C4H6O2+	☐	☐
1-penten-3-ol	O2+	2.4E-9	13	86	C5H10O+	☐	☐
cis-2-penten-1-ol	O2+	2.7E-9	9	86	C5H10O+	☐	☐
heptanal	O2+	3.2E-9	12	86	C5H10O+	☐	☐
methyl hexanoate	O2+	1.7E-9	17.5	87	C5H11O+	☐	☐
2-phenylethanol	O2+	2.4E-9	20	91	C7H7+	☐	☐
benzyl alcohol	O2+	2.3E-9	5	91	C7H7+	☐	☐
(R)-limonene	O2+	2.2E-9	9	92	C7H8+	☐	☐
(Z)-beta-ocimene	O2+	1.9E-9	9	92	C7H8+	☐	☐
2-phenylethanol	O2+	2.4E-9	75	92	C7H8+	☐	☐
benzyl alcohol	O2+	2.3E-9	5	92	C7H8+	☐	☐

		rate	ratio (%)	(m/z)			
myrcene	O2+	2.2E-9	69	92	C7H8+	☐	☐
(R)-limonene	O2+	2.2E-9	29	93	C7H9+	☐	☐
(Z)-beta-ocimene	O2+	1.9E-9	45	93	C7H9+	☐	☐
D-carvone	O2+	2.7E-9	10	93	C7H9+	☐	☐
linalool	O2+	2.5E-9	30	93	C7H9+	☐	☐
linalyl acetate	O2+	2.9E-9	10	93	C7H9+	☐	☐
myrcene	O2+	2.2E-9	5	93	C7H9+	☐	☐
(R)-limonene	O2+	2.2E-9	12	94	C7H10+	☐	☐
(Z)-beta-ocimene	O2+	1.9E-9	5	94	C7H10+	☐	☐
myrcene	O2+	2.2E-9	5	94	C7H10+	☐	☐
heptanal	O2+	3.2E-9	22	96	C7H12+	☐	☐
linalool	O2+	2.5E-9	10	96	C7H12+	☐	☐
nonanal	O2+	1.5E-9	11	96	C7H12+	☐	☐
hexanal	O2+	2.0E-9	5	97	C6H9O+	☐	☐
(E)-2-hexenal	O2+	3.7E-9	20	98	C6H10O+	☐	☐
nonanal	O2+	1.5E-9	18	98	C6H10O+	☐	☐
1-octen-3-ol	O2+	2.3E-9	6	99	C6H11O+	☐	☐
methyl hexanoate	O2+	1.7E-9	20	99	C6H11O+	☐	☐
(E)-2-hexen-1-ol	O2+	2.9E-9	5	100	C6H12O+	☐	☐
3-hexen-1-ol	O2+	2.9E-9	5	101	C6H12O.H+	☐	☐
3-hexen-1-ol	O2+	2.9E-9		119	C6H12O.H+.H2O	☐	☐
methyl hexanoate	O2+	1.7E-9	7.5	101	C6H13O+	☐	☐
benzaldehyde	O2+	2.8E-9	30	105	C7H5O+	☑	☑
benzaldehyde	O2+	2.8E-9	70	106	C7H6O+	☐	☐
D-carvone	O2+	2.7E-9	10	106	C8H10+	☐	☐
benzyl alcohol	O2+	2.3E-9	25	107	C7H7O+	☐	☐
(R)-limonene	O2+	2.2E-9	11	107	C8H11+	☐	☐
(Z)-beta-ocimene	O2+	1.9E-9	7	107	C8H11+	☐	☐
benzyl alcohol	O2+	2.3E-9	45	108	C7H8O+	☐	☐
6-methyl-5-hepten-2-one	O2+	2.5E-9	65	108	C8H12+	☐	☐
D-carvone	O2+	2.7E-9	25	108	C8H12+	☐	☐
1-octen-3-ol	O2+	2.3E-9	4	110	C8H14+	☐	☐
octanal	O2+	2.8E-9	20	110	C8H14+	☐	☐
2-heptanone	O2+	3.2E-9	20	114	C7H14O+	☐	☐
nonanal	O2+	1.5E-9	6	114	C7H14O+	☐	☐
linalool	O2+	2.5E-9	10	121	C8H9O+	☐	☐
(R)-limonene	O2+	2.2E-9	13	121	C9H13+	☐	☐
(Z)-beta-ocimene	O2+	1.9E-9	18	121	C9H13+	☐	☐
myrcene	O2+	2.2E-9	6	121	C9H13+	☐	☐
2-phenylethanol	O2+	2.4E-9	5	122	C8H10O+	☐	☐
beta-cyclocitral	O2+	3.0E-9	15	123	C9H15+	☐	☐
lemonol	O2+	2.5E-9	10	123	C9H15+ or C8H11O	☐	☐
nonanal	O2+	1.5E-9	15	124	C9H16+	☐	☐
6-methyl-5-hepten-2-one	O2+	2.5E-9	35	126	C8H14O+	☐	☐
(R)-limonene	O2+	2.2E-9	11	136	C10H16+	☐	☐
(Z)-beta-ocimene	O2+	1.9E-9	6	136	C10H16+	☐	☐
lemonol	O2+	2.5E-9	15	136	C10H16+	☐	☐
myrcene	O2+	2.2E-9	3	136	C10H16+	☐	☐
linalool	O2+	2.5E-9	20	137	C10H17+	☐	☐
beta-cyclocitral	O2+	3.0E-9	35	137	C9H13O+	☐	☐
methyl salicylate	O2+	2.7E-9	40	138	C7H6O3+	☐	☐
decane	O2+	2.0E-9	35	142	C10H22+	☑	☑
D-carvone	O2+	2.7E-9	35	150	C10H14O+	☑	☑
beta-cyclocitral	O2+	3.0E-9	50	152	C10H16O+	☐	☐
methyl salicylate	O2+	2.7E-9	60	152	C8H8O3+	☐	☐
dodecane	O2+	1.5E-9	40	170	C12H26+	☑	☑
beta-ionone	O2+	2.5E-9	75	177	C12H17O+	☑	☑
beta-ionone	O2+	2.5E-9	25	192	C13H20O+	☐	☐

H3O+
NO+
O2+
Warnings

Primary product C3H7O+ of 2,4-heptadienal with H3O+ is not calculated
Primary product C8H9+ of 2-phenylethanol with H3O+ is not calculated
Primary product C10H14O.H+ of D-carvone with H3O+ is not calculated
Primary product C8H17O+ of octanal with H3O+ is not calculated
Compound (R)-limonene is not calculated
Compound (Z)-beta-ocimene is not calculated
Compound 1-octen-3-ol is not calculated
Compound 1-penten-3-ol is not calculated
Compound 3-hexen-1-ol is not calculated
Compound cis-2-penten-1-ol is not calculated
Compound hexanal is not calculated
Compound myrcene is not calculated
Compound nonanal is not calculated
Compound pentanal is not calculated

Table S3: GC-FID peak area intensities of the 48 compounds with the sample correspondence

Sample	Country	Region	Color
1	Nepal	Ilam Valley	White
2	Nepal	Ilam Valley	White
3	Nepal	Ilam Valley	White
4	China	Zhejiang	Green
5	China	Zhejiang	Green
6	China	Zhejiang	Green
7	Japan	Kyushu	Green
8	Japan	Kyushu	Green
9	China	Jiangsu	Black
10	China	Jiangsu	Black
11	China	Jiangsu	Black
12	India	Darjeeling	Black
13	India	Darjeeling	Black
14	India	Darjeeling	Black
15	Taiwan	Monts Ali	Black
16	Taiwan	Monts Ali	Black
17	Vietnam	Lai Chau	Oolong
18	Vietnam	Lai Chau	Oolong
19	Vietnam	Lai Chau	White
20	Vietnam	Lai Chau	Black
21	Vietnam	Lao Cai	Green
22	China	Yunnan	Dark
23	China	Yunnan	Dark
24	China	Yunnan	Dark
25	Sri Lanka	Ratnapura	Black
26	Sri Lanka	Matara	Black

Compound	RT	1	2	3	4	5	6	7	8	9	10	11	12	13	14	15	16	17	18	19	20	21	22	23	24	25	26
**Acid acetic**	5,034	0,00	1 081 835,75	573 751,57	493 750,75	0,00	0,00	193 037,27	233 755,26	568 685,76	428 885,96	180 143,25	835 098,85	6 352 313,82	762 129,37	0,00	0,00	0,00	0,00	0,00	0,00	0,00	0,00	0,00	0,00	0,00	0,00
**1-penten-3-ol**	6,09	0,00	0,00	0,00	0,00	347 843,37	100 434,23	51 279,02	135 364,07	208 312,51	317 700,81	71 144,39	74 528,00	496 344,91	90 827,91	61 990,75	324 986,91	247 331,39	150 811,62	79 080,96	91 574,47	96 791,24	62 470,01	36 751,81	88 105,45	58 419,73	54 202,97
**Pentanal**	6,34	0,00	0,00	0,00	65 900,70	0,00	0,00	87 426,55	114 940,08	0,00	0,00	0,00	79 558,38	798 503,71	0,00	18 809,80	50 534,47	77 828,47	148 332,82	37 739,54	40 611,35	0,00	0,00	0,00	0,00	0,00	0,00
**1-Pentanol**	7,634	0,00	0,00	0,00	26 013,46	64 804,65	37 500,04	41 680,28	59 168,50	83 479,06	117 755,54	51 002,82	62 462,90	633 052,80	0,00	38 220,87	0,00	250 908,02	40 038,57	0,00	114 810,14	0,00	0,00	45 391,81	38 686,80	23 878,17	42 819,09
**2-penten-1-ol**	7,69	0,00	0,00	0,00	30 698,02	126 074,14	38 058,67	32 673,44	109 294,06	103 826,75	1 431 961,15	96 971,15	69 006,97	1 954 666,37	0,00	0,00	133 350,43	151 172,63	33 020,97	83 661,40	205 923,09	80 119,96	53 518,42	82 186,96	51 454,98	132 677,01	110 126,15
**3-penten-2-one, 4-methyl**	8,244	0,00	0,00	0,00	0,00	27 264,07	26 087,22	31 677,74	22 517,52	0,00	0,00	0,00	0,00	0,00	0,00	0,00	0,00	0,00	1 558 101,04	0,00	0,00	32 690,10	20 979,41	16 953,98	48 458,01	0,00	0,00
**Hexanal**	8,298	156 589,41	194 255,90	280 232,87	40 873,35	66 153,69	29 392,23	22 419,22	36 177,03	92 422,68	1 071 742,19	84 981,89	203 180,60	3 883 864,01	246 329,30	269 600,16	396 823,19	1 064 641,31	117 714,46	349 303,60	756 436,73	58 291,57	42 396,41	30 790,47	31 701,89	68 325,10	74 844,80
**2-hexenal**	9,49	83 800,14	473 857,55	547 306,46	288 443,77	443 008,73	226 276,44	0,00	0,00	0,00	0,00	0,00	0,00	0,00	0,00	11 341,14	18 105,86	63 434,26	97 155,47	118 053,31	107 876,28	17 144,79	0,00	0,00	0,00	43 127,53	62 473,49
**3-hexen-1-ol**	9,54	550 265,63	0,00	0,00	0,00	0,00	0,00	195 220,90	178 161,26	633 308,65	492 379,93	300 448,33	771 197,47	494 349,26	789 611,18	0,00	0,00	42 709,91	116 257,00	72 638,67	58 053,18	38 687,10	0,00	0,00	0,00	283 361,13	206 645,82
**2-hexen-1-ol**	9,77	0,00	0,00	0,00	0,00	0,00	0,00	0,00	0,00	0,00	0,00	0,00	0,00	0,00	0,00	0,00	0,00	91 074,23	0,00	26 128,07	48 149,07	0,00	0,00	0,00	0,00	85 474,91	45 930,44
**1-Hexanol**	9,844	0,00	0,00	0,00	0,00	0,00	0,00	0,00	0,00	0,00	0,00	0,00	0,00	0,00	0,00	0,00	0,00	0,00	0,00	63 652,19	0,00	0,00	0,00	0,00	0,00	0,00	0,00
**pentanoic acid**	10,25	81 908,15	0,00	0,00	0,00	0,00	0,00	0,00	0,00	0,00	0,00	0,00	0,00	0,00	0,00	0,00	0,00	0,00	0,00	0,00	0,00	0,00	0,00	0,00	0,00	0,00	0,00
**2-heptanon**	10,36	0,00	0,00	0,00	0,00	0,00	0,00	0,00	0,00	0,00	0,00	0,00	0,00	0,00	0,00	27 874,53	0,00	92 330,75	0,00	27 099,42	45 150,95	0,00	0,00	0,00	0,00	0,00	0,00
**Heptanal**	10,71	0,00	0,00	0,00	82 164,82	77 720,16	78 159,02	32 923,37	28 588,22	0,00	0,00	0,00	0,00	0,00	0,00	19 667,77	20 319,45	108 157,68	0,00	57 944,52	70 862,28	0,00	15 118,76	42 464,61	15 074,85	0,00	0,00
**butyrolactone**	10,98	0,00	0,00	0,00	0,00	0,00	0,00	32 802,65	65 210,50	105 967,10	89 504,92	65 051,58	87 374,09	2 376 166,78	76 999,39	0,00	0,00	59 542,16	0,00	0,00	0,00	0,00	0,00	0,00	0,00	44 025,63	56 627,02
**Butanoic acid, 4-hydroxy**	11,01	0,00	0,00	0,00	46 270,45	33 766,92	16 525,75	0,00	0,00	0,00	0,00	0,00	0,00	0,00	0,00	0,00	0,00	0,00	0,00	0,00	0,00	0,00	0,00	0,00	0,00	0,00	0,00
**Hexanoic acid, methyl ester**	11,31	0,00	0,00	0,00	0,00	0,00	0,00	0,00	0,00	47 347,35	206 399,02	0,00	0,00	0,00	0,00	0,00	0,00	16 683,84	42 441,48	0,00	0,00	0,00	0,00	0,00	0,00	0,00	0,00
**Benzaldehyde**	12,61	566 510,57	551 875,18	414 541,40	26 015,36	43 426,81	23 866,55	0,00	0,00	392 280,25	466 154,55	317 211,78	202 259,30	609 775,65	268 630,85	11 304,80	43 449,13	369 002,91	252 471,80	252 563,59	441 517,90	80 544,83	36 302,60	13 695,88	37 710,51	103 715,05	195 577,33
**1-Octen-3-ol**	12,97	118 163,09	141 289,41	237 682,42	289 928,89	132 635,97	25 655,00	0,00	0,00	0,00	0,00	0,00	108 594,44	651 675,37	135 726,03	0,00	0,00	73 078,47	137 652,88	20 556,68	32 427,05	0,00	0,00	84 748,83	157 772,07	0,00	0,00
**5-hepten-2-one, 6-methyl**	13,1	101 848,56	111 701,81	160 003,08	0,00	131 928,78	60 416,01	70 500,36	94 739,35	181 921,26	279 846,94	20 885,87	107 786,09	371 766,19	161 784,32	45 471,49	71 221,12	153 966,31	296 405,54	105 655,54	119 783,26	65 097,03	0,00	26 998,40	51 710,43	26 525,14	117 229,97
**Hexanoic acid**	13,24	99 771,90	0,00	0,00	0,00	0,00	0,00	0,00	0,00	0,00	0,00	0,00	146 446,92	1 697 857,14	150 404,96	49 980,97	105 629,43	200 119,96	0,00	0,00	250 105,44	0,00	0,00	0,00	0,00	0,00	0,00
**b-myrcene**	13,262	0,00	0,00	0,00	55 524,37	0,00	0,00	0,00	0,00	0,00	0,00	0,00	0,00	0,00	0,00	0,00	0,00	311 392,91	0,00	0,00	0,00	182 457,57	167 900,23	118 119,14	144 479,86	100 743,75	364 172,33
**Furan, 2-pentyl**	13,3	0,00	0,00	0,00	0,00	102 616,54	50 193,81	77 763,33	248 698,23	127 148,23	478 166,72	171 887,59	115 258,85	598 802,18	161 779,03	177 041,71	53 709,28	81 903,68	324 814,96	602 212,88	0,00	76 427,87	73 818,53	28 518,11	0,00	50 256,93	46 162,46
**Decane**	13,54	0,00	0,00	0,00	0,00	49 502,62	26 356,54	35 508,97	75 234,07	70 606,10	53 502,99	0,00	101 490,80	7 184 623,72	801 194,11	165 316,62	1 064 306,45	94 261,40	204 723,52	37 315,81	83 592,08	69 739,34	78 357,96	22 411,41	61 089,73	50 314,71	2 143 371,39
**Octanal**	13,61	0,00	0,00	0,00	0,00	706 446,45	626 221,57	0,00	0,00	0,00	0,00	0,00	0,00	0,00	0,00	0,00	0,00	88 893,07	0,00	36 756,34	40 151,78	0,00	0,00	7 705,84	0,00	0,00	0,00
**2,4-heptadienal**	13,86	0,00	0,00	0,00	0,00	159 499,73	184 674,43	0,00	0,00	0,00	0,00	0,00	0,00	0,00	0,00	0,00	0,00	1 559 641,27	139 513,52	0,00	55 055,95	0,00	0,00	0,00	0,00	0,00	0,00
**D-limonene**	14,42	281 179,61	288 865,10	417 734,86	82 824,67	0,00	0,00	259 230,17	277 811,72	0,00	0,00	0,00	577 090,97	1 034 805,37	0,00	850 020,59	94 532,44	667 649,12	1 321 989,13	508 967,10	1 064 007,19	1 660 533,23	1 505 659,44	513 583,94	855 858,17	2 294 811,97	232 783,85
**Benzyl alcohol**	14,5	967 105,49	920 531,43	810 965,44	938 786,90	0,00	0,00	195 793,70	266 041,40	164 748,01	1 288 026,83	293 724,60	479 645,42	3 083 281,29	231 561,69	0,00	0,00	186 547,14	298 085,54	108 949,46	270 805,55	0,00	0,00	0,00	64 912,53	0,00	0,00
**b-cis-o-cimene**	14,75	0,00	0,00	0,00	0,00	0,00	0,00	0,00	0,00	1 213 843,68	648 412,65	1 547 666,14	0,00	0,00	0,00	0,00	0,00	390 950,86	0,00	339 980,35	165 252,68	0,00	65 737,87	54 646,15	85 515,44	134 135,88	155 159,53
**3,5-octadien-2-one**	15,3	145 607,95	0,00	0,00	0,00	0,00	0,00	63 297,95	142 816,46	0,00	0,00	0,00	235 556,81	1 641 100,89	294 668,84	0,00	0,00	0,00	556 449,41	0,00	150 478,00	0,00	0,00	0,00	0,00	0,00	0,00
**Linalool oxide**	15,41	382 828,60	780 714,14	863 834,09	0,00	0,00	0,00	0,00	0,00	1 099 922,62	678 934,22	0,00	678 998,50	3 127 575,40	905 226,61	63 923,86	51 378,34	237 573,95	0,00	272 821,33	603 161,99	0,00	0,00	0,00	0,00	0,00	0,00
**trans linalool oxide**	15,77	988 806,06	1 758 644,85	2 162 341,51	118 903,71	0,00	0,00	0,00	0,00	3 065 687,23	1 889 647,82	480 002,08	1 897 125,78	6 489 466,43	2 365 142,34	32 074,01	154 038,85	329 223,05	0,00	574 237,18	700 473,82	0,00	0,00	0,00	94 812,29	153 823,24	1 707 570,58

**Linalool**	16,01	7 191 440,53	9 507 894,49	13 673 028,09	488 885,81	614 860,13	491 551,98	102 416,62	88 346,44	3 649 866,29	2 158 835,66	632 018,46	5 809 129,68	21 125 662,92	7 314 490,98	313 779,43	47 852,44	857 387,77	404 440,26	1 608 639,48	874 085,48	946 552,35	582 721,53	670 714,13	659 823,12	1 976 319,69	210 049,53
**Nonanal**	16,1	0,00	0,00	0,00	88 214,66	127 563,43	220 896,35	75 983,92	82 329,68	0,00	0,00	0,00	0,00	0,00	0,00	0,00	23 194,43	504 366,70	592 259,36	589 056,28	493 744,85	135 225,69	123 850,55	125 882,61	122 521,01	0,00	0,00
**Phenylethyl alcohol**	16,4	0,00	0,00	0,00	955 978,96	1 033 712,76	855 276,63	491 807,26	972 177,28	1 083 518,04	1 294 167,83	544 904,23	555 531,75	4 109 297,07	480 081,82	0,00	0,00	0,00	0,00	0,00	0,00	0,00	0,00	0,00	0,00	0,00	0,00
**Undecane, 3-methyl**	17,48	0,00	0,00	0,00	141 096,04	153 900,42	97 722,29	0,00	0,00	0,00	0,00	0,00	0,00	0,00	0,00	18 306,32	0,00	33 139,46	696 194,00	0,00	0,00	441 131,21	373 241,35	0,00	0,00	0,00	0,00
**trans-linalool 3,7 oxide**	17,56	0,00	0,00	0,00	119 109,43	0,00	0,00	0,00	0,00	783 924,81	427 171,34	275 411,11	0,00	0,00	0,00	194 949,25	0,00	159 970,48	197 563,38	166 969,34	266 852,96	222 175,43	288 879,35	0,00	0,00	0,00	0,00
**Methyl salicylate**	18,09	0,00	434 660,15	625 139,68	0,00	0,00	0,00	0,00	0,00	1 119 518,37	496 081,09	405 536,65	888 700,41	5 626 814,23	1 645 455,25	0,00	0,00	0,00	0,00	0,00	0,00	0,00	0,00	0,00	0,00	0,00	0,00
**dodecane**	18,04	0,00	0,00	0,00	0,00	0,00	0,00	0,00	0,00	0,00	0,00	0,00	0,00	0,00	0,00	57 251,78	223 631,60	145 261,73	298 009,57	67 124,00	134 368,69	0,00	0,00	35 797,65	43 659,57	228 357,46	91 649,24
**b-cyclocitral**	18,55	0,00	0,00	0,00	99 177,58	174 707,64	89 369,44	510 476,71	524 048,53	0,00	0,00	0,00	129 627,03	1 957 008,49	168 652,62	14 161,13	67 769,28	0,00	204 575,39	0,00	0,00	0,00	0,00	63 109,99	54 956,07	58 417,37	269 075,45
**Butanoic acid, 2-methyl-, hexyl ester**	18,66	0,00	0,00	0,00	0,00	0,00	0,00	0,00	0,00	0,00	0,00	0,00	0,00	0,00	0,00	0,00	0,00	0,00	298 845,55	0,00	61 500,09	0,00	0,00	0,00	0,00	0,00	0,00
**Linalyl acetat**	18,922	0,00	0,00	0,00	186 811,49	0,00	0,00	0,00	0,00	0,00	0,00	0,00	344 930,21	4 209 384,70	374 175,50	0,00	0,00	624 304,84	0,00	0,00	0,00	0,00	0,00	183 584,19	240 087,01	169 363,95	65 150,73
**D-Carvone**	18,976	0,00	0,00	0,00	0,00	0,00	0,00	0,00	0,00	0,00	0,00	0,00	0,00	0,00	0,00	0,00	0,00	459 898,04	0,00	0,00	0,00	0,00	0,00	147 378,85	333 658,35	70 711,95	45 671,10
**geraniol**	18,96	605 628,42	661 275,33	1 171 219,84	0,00	0,00	0,00	0,00	0,00	1 120 998,27	326 397,53	981 123,65	416 249,95	1 095 143,32	684 756,74	0,00	0,00	0,00	133 535,45	1 538 826,53	590 374,38	0,00	0,00	0,00	0,00	0,00	0,00
**Anethole**	19,68	0,00	0,00	0,00	0,00	0,00	0,00	0,00	0,00	0,00	0,00	0,00	0,00	0,00	0,00	0,00	0,00	213 539,21	0,00	1 368 524,33	318 391,82	795 753,66	339 623,20	96 669,06	301 919,31	77 646,85	725 476,35
**a-longipinene**	20,85	0,00	0,00	0,00	0,00	0,00	0,00	0,00	0,00	0,00	0,00	0,00	0,00	0,00	0,00	24 185,93	26 085,41	0,00	0,00	0,00	0,00	0,00	0,00	0,00	0,00	44 963,65	89 979,05
**tetradecane**	21,282	0,00	0,00	0,00	26 954,54	17 393,39	0,00	136 793,19	147 518,95	55 130,51	184 382,03	0,00	0,00	0,00	0,00	35 323,36	101 479,73	0,00	62 623,72	0,00	0,00	0,00	54 369,53	0,00	45 926,70	25 080,45	722 430,40
**b-Ionone**	22,52	212 964,78	0,00	0,00	68 046,14	106 250,07	68 253,99	78 268,77	175 887,06	63 592,14	59 708,90	23 746,44	228 677,51	4 327 581,94	201 375,58	34 712,40	47 084,66	99 531,47	88 461,77	140 109,93	106 926,54	202 315,25	137 016,25	0,00	0,00	55 550,65	279 727,96
**b-Ionone epoxide**	22,56	0,00	0,00	0,00	0,00	20 362,98	12 771,30	33 457,62	59 627,01	0,00	0,00	0,00	0,00	0,00	0,00	0,00	0,00	52 073,50	17 707,09	0,00	0,00	0,00	0,00	37 754,27	134 716,63	0,00	0,00
**caffeine**	26,8	0,00	0,00	0,00	47 148,73	66 187,01	59 038,33	110 157,23	116 108,97	84 578,38	62 159,07	111 287,05	0,00	1 006 566,08	0,00	20 184,57	36 071,65	33 265,79	19 502,37	0,00	14 557,70	45 155,93	29 770,78	26 379,74	26 951,97	40 202,52	0,00
